# Emerging roles of long non-coding RNA in root developmental plasticity and regulation of phosphate homeostasis

**DOI:** 10.3389/fpls.2015.00400

**Published:** 2015-06-09

**Authors:** Jeremie Bazin, Julia Bailey-Serres

**Affiliations:** ^1^Department of Botany and Plant Sciences, Center for Plant Cell Biology, University of California, Riverside, CA, USA; ^2^Saclay Plant Science, Institut des Sciences du Végétal, Centre National de la Recherche Scientifique, Gif-sur-Yvette, France

**Keywords:** long non-coding RNA, phosphate homeostasis, root development, chromatin loop, alternative splicing, RNA sequencing, translation regulation

## Abstract

Long non-coding RNAs (lncRNAs) have emerged as important regulators of gene expression in a variety of biological process and in multiple species. In plants, they are transcribed by different RNA polymerases and show diverse structural features. With the aid of next-generation sequencing technologies, a large number of lncRNA have been identified in model plants as well as in crops. This review focuses on the demonstration that lncRNAs control root system architecture, notably in response to phosphate availability, through regulation of transcription, alternative splicing, microRNA activity, messenger RNA stability and translation, illustrating remarkable diversity in their roles in regulating developmental plasticity.

## Introduction

Plants are genetically predisposed to continuously adapt their cellular metabolism and development to changes in the environment. This plasticity is key to the evolutionary success of plants in diverse ecosystems but has not always been selected for during the domestication and improvement of crops. Given the need for crops to be more resilient to environmental variation and less reliant on nutrient inputs, comprehension of molecular mechanisms controlling plant developmental plasticity in response to variations in the environment could aid improvement of crop yields in the future. Approximately 70% of the cultivated lands have limited levels of inorganic phosphate (Pi), requiring phosphate inputs as fertilizer or else limiting agricultural production. Low Pi availability in the soil results in Pi deficiency that triggers a cascade of developmental responses, which reprogram the root system architecture. This developmental plasticity allows roots to favor exploration of the shallow part of the soil, where phosphate tends to be more abundant and thus maximizing its uptake. This process is under the control of spatiotemporal-regulated gene expression networks (Misson et al., 2005; Secco et al., 2013). These determine the balance between cell division and elongation driving apical growth and cell differentiation processes that control root branching through the formation of lateral roots. LncRNAs are a diverse class of RNA molecules generated from multiple RNA polymerases that have emerged as important players in the regulation of gene expression. In particular, their role in the regulation of transcription has been actively explored in animals as well as in plants, albeit to a lesser extent. Specifically, lncRNAs play an important role in chromatin remodeling by interacting with chromatin modification complexes involved in gene silencing (Rinn and Chang, 2012), such as the Polycomb Repressive Complexes 1 and 2 (PRC1, PRC2). Examples in plants include the vernalization-regulated lncRNAs *COOLAIR* and *COLDAIR* (Swiezewski et al., 2009; Heo and Sung, 2011), which interact with PRC2 and mediate the deposition of the repressive chromatin mark, H3K27me3 at the floral repressor gene *FLOWERING LOCUS C* (*FLC*). Sequencing-by-synthesis technologies has revealed lncRNAs as a significant component of the transcriptome in a number of plant species (Geisler and Coller, 2013), including the models *Arabidopsis thaliana* and rice (*Oryza sativa*; Kim and Sung, 2012). So far, the functions of only a small number of lncRNAs are known. These include the above-mentioned roles of lncRNAs in transcriptional regulation through chromatin remodeling, pre-mRNA splicing (Bardou et al., 2014) and mRNA translation (Jabnoune et al., 2013). Recent data indicate that lncRNAs contribute to the regulation of developmental plasticity of root systems, particularly in response to a deficiency in Pi.

This review considers the remarkably diverse roles lncRNAs play in environmental regulation of root system architecture.

## The Long Non-coding RNA “World” in Plants

When eukaryotic genomes were first sequenced, the proportion encoding protein coding gene regions was found to represent only a small fraction of the total genome. However, unbiased, high throughput transcriptome analysis technologies such as deep sequencing and tilling arrays revealed extensive transcription in intergenic regions (Birney et al., 2007). Non-protein coding RNA molecules include structural and housekeeping RNA (ribosomal RNA, tRNA, small nucleolar RNAs, small nuclear RNA), as well as a large and heterogeneous class of regulatory RNAs. Among these are, small non-coding RNAs including microRNAs and small interfering RNAs, which have been actively studied for their roles in gene silencing (Kestrel and Chen, 2013). More recently, studies in plants and animals have drawn attention to lncRNAs (>200 nt). These can be sub-classified according to their genomic origin and/or their orientation compared to neighboring protein-coding transcripts. Long intergenic or intronic RNAs (lincRNAs) are transcribed from intergenic or intronic regions. Natural antisense transcripts (NATs) are non-coding RNAs that are complementary to protein-coding transcripts. These originate from the reverse strand of sense coding regions (*cis*-NATs) or from distinct genomic loci (*trans*-NATs; Cech and Steitz, 2014).

Most of lncRNAs identified in eukaryotes are likely to be transcribed by RNA polymerase II (pol II) in a process in plants involving the eukaryotic multi-subunit protein complex Mediator (Kim et al., 2011). They also harbor characteristic features of mRNAs such a 5′-^7m^GTP-cap and 3′-polyadenylated (polyA) tail. Plant specific RNA polymerases IV and V (pol IV and pol V), which evolved from pol II (Vaughn and Martienssen, 2005), also act in lncRNA biogenesis and function to create a more complex lncRNA population. For example, pol IV is required for the biogenesis heterochromatic siRNAs (Zhang et al., 2007), whereas polV transcribes lncRNAs, which act as a scaffold to recruit the DNA methylation machinery at genomic loci complementary to heterochromatic siRNAs (Wierzbicki et al., 2008).

Pol IV-dependent siRNA precursors in plants were elusive until recently because of their instability and low abundance due to their silencing at the chromatin level involving high cytosine methylation. By use of a combination of RNA-sequencing approaches with mutants affected in pol IV-dependent transcription, Li et al. (2014) identified more the 20,000 pol IV-dependent lncRNAs. This study also revealed that lncRNAs produced by pol IV possess a 5′ monophosphate and lack a 3′-poly(A) tail, distinguishing them from lncRNAs synthesized by pol II. By contrast to pol II and pol IV products, pol V transcripts are 5′-triphosphorylated or ^7m^GTP-capped, but do not have 3′-poly(A) tails (Li et al., 2014). A comparison of datasets of small RNAs and lncRNA loci uncovered that a large proportion lncRNAs are not templates of small RNA biogenesis, suggesting they function in small RNA–independent pathways (Liu et al., 2012a).

The use of microarrays that tiled the complete genome of *Arabidopsis* uncovered nearly 6,500 lincRNAs that accumulate across diverse organs of *Arabidopsis*, of which 2,708 were reconfirmed in a subsequent RNA-seq analysis (Liu et al., 2012a). Using a similar approach, Wang et al. (2014) found that ∼70% of *Arabidopsis* protein-coding genomic loci encode potential NATs. Although a portion of the recently identified lncRNAs could possibly be by-products of mRNA transcription, a significant number of plant lncRNAs exhibit properties suggesting their synthesis or accumulation is controlled. These include evidence of tissue-specific expression (Sugiyama et al., 2007; Jiao and Meyerowitz, 2010; Liu et al., 2012a; Wang et al., 2014), sub-cellular localization (Campalans et al., 2004) and/or enhancement or repression in response to environmental stress (Franco-Zorrilla et al., 2007; Ben Amor et al., 2009; Heo and Sung, 2011). This argues that lncRNAs are functionally relevant rather than remnants of transcriptional processes (i.e., transcriptional noise).

## Functions of lncRNAs in Root Development

Recent studies have begun to unravel the role of lncRNAs in the control of root development. Through bioinformatic analysis of full length *Arabidopsis* cDNAs, Ben Amor et al. (2009) identified 76 lncRNA, including 42 with increased abundance in roots and responsive to at least one of the following stresses: phosphate starvation, salt stress or drought stress. The ectopic overexpression of two of these lncRNAs affected growth and cell differentiation in roots of *Arabidopsis* (Ben Amor et al., 2009). This intriguing observation led to deeper functional analyses. Recently, the function of *APOLO* lncRNA (*AUXIN REGULATED PROMOTER LOOP RNA*, previously known as *NPC34*; Ben Amor et al., 2009) was deciphered, revealing a role in chromatin structure of a neighboring locus (Ariel et al., 2014).

**FIGURE 1:**
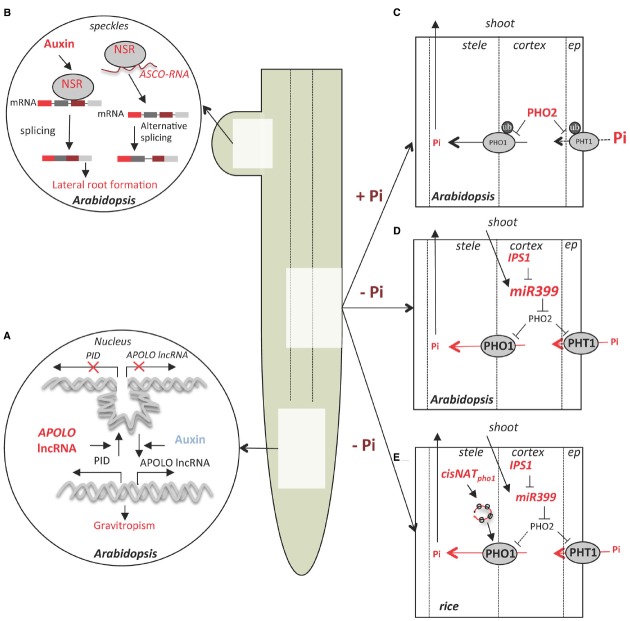
**lncRNA control of root development, Pi uptake and homeostasis in *Arabidopsis* and rice. (A)** *APOLO* lncRNA is induced by auxin in *Arabidopsis* roots and controls the dynamics of a chromatin loop at the *PINOID* (*PID*) promoter. This mechanism regulates the dynamics of *PID* transcription and contributes to gravitropism (Ariel et al., 2014). **(B)** Control of auxin-dependent lateral root formation in *Arabidopsis* by splicing regulators Nuclear Speckle RNA-binding proteins (NSRs) involve regulation by lncRNA *ASCO* which compete with NSRs for pre-mRNA biding and regulate alternative splicing of a subset of genes (Bardou et al., 2014). **(C)** Under Pi sufficiency (+Pi), the ubiquitin conjugating enzyme PHO2 mediates the ubiquitinylation (ub) of several members of the epidermis-localized high-affinity phosphate transporters family (PHT1) as well as PHO1, involved in Pi translocation to the xylem (Liu et al., 2012b; Huang et al., 2013), leading to a reduced Pi uptake and transport. **(D)** Under Pi deficiency (-Pi) *miR399* is transported through the phloem from the shoot to the root where it induces *PHO2* mRNA cleavage and consequently to the stabilization of the PHO1 and PHT1 transporters. At the same time, induction of the lncRNA *IPS1* dampens *miR399* activity and allows dynamic regulation of Pi homeostasis (Franco-Zorrilla et al., 2007). **(E)** In rice, in addition to the mechanisms described in *Arabidopsis*, low Pi conditions induce transcription if a *cis*-NAT to *PHO1;2* (cisNAT_pho1;2_), which stimulates *PHO1;2* mRNA translation; PHO1 accumulation increases Pi transport from the root to shoot (Jabnoune et al., 2013).

*APOLO* is located 5,148 bp upstream of *PINOID* (*PID*), which encodes a regulatory kinase that determines the polar localization of the auxin transporter PIN-FORMED 2 (PIN2) in root cells (Huang et al., 2010). *PID* and *APOLO* are co-regulated and transiently induced by application of exogenous auxin (Figure 1A). The downregulation of *APOLO* lncRNA production corresponds with reduced *PID* mRNA accumulation. Plants with *APOLO* transcription reduced by RNAi display altered primary root growth and a delayed gravitropism response, similar to *pid* mutants. In addition, chromatin analysis of the *PID-APOLO* genomic region demonstrated the presence of a chromatin loop encompassing the *PID* promoter, which is maintained by DNA methylation sustained by pol IV/pol V transcription of *APOLO* lncRNA.

By use of a system that allows synchronization of the formation of lateral organs in roots, the authors showed that auxin recruits the DNA Demethylation Machinery (DDM) at the *PID* promoter in root cells. This opens the chromatin loop and allows Pol II dependent transcription of *PID* mRNA and *APOLO* lncRNA. The accumulation of *APOLO* lncRNA promotes recruitment of LIKE HETEROCHROMATIN PROTEIN 1 (LHP1), a member of the Polycomb Repressive Complex 1 (PRC1), whereas pol IV/V transcription of *APOLO* triggers its methylation, mediating loop closure and thus downregulation of *PID* transcription by pol II. Hence, this mechanism fine-tunes *PID* promoter activity and could enable oscillation of gene expression in roots in response to auxin.

Another mechanism of lncRNA action was exposed in a functional analysis of the nuclear speckle RNA-binding proteins (NSRs) of *Arabidopsis* (Bardou et al., 2014). The paralogs *AtNSRa* and *AtNSRb* are expressed specifically in root meristems and lateral roots. The *nsra nsrb* double mutant has fewer and shorter lateral roots and is less sensitive to the stimulation of lateral root growth by auxin. At the molecular level, AtNSRs modulate auxin-dependent alternative splicing (AS) of a specific subset of genes which are involved in the control of lateral root initiation (Figure 1B). In a prior study of *Medicago truncatula*, an ortholog of AtNSR (MtRBP1/MtNSR) was identified in a screen for proteins that bind the short open reading frame mRNA called *ENOD40*, a highly structured long RNA (Compalans et al., 2004). This study found *ENOD40* overexpression caused the relocalization of MtRBP1/MtNSR from nuclear speckles to the cytoplasm. Interestingly, this phenomenon was recapitulated when *ENOD40* was co-expressed with AtNSR in tobacco cells. An *Arabidopsis* lncRNA that binds AtNSRs called *ASCO* lncRNA (AS competitor long non-coding RNA) was identified (Bardou et al., 2014). The strong similarity in phenotype of *ASCO* lncRNA overexpressing lines and the *nsra nsrb* double mutant suggests *ASCO* may negatively regulate NSR activity. Although *ASCO* overexpression did not induce NSR relocalization to the cytoplasm, the authors showed that this lncRNA is able to modulate NSR dependent AS events through direct binding to NSRs and displacement from its endogenous mRNA target. In other words, *ASCO* RNA can mimic the endogenous alternatively spliced transcript to hijack the splicing machinery and regulate rapid changes in AS pattern during development. The large number of newly identified lncRNAs that are regulated by different environmental clues in roots suggests these transcripts contribute to the plasticity of the root system architecture.

## Roles of lncRNAs in Control of Phosphate (Pi) Homeostasis

Plants have evolved an array of physiological and biochemical responses to cope with dynamics in the moisture, nutrients and microbiome of the soil. This is particularly striking when plants experience limiting Pi availability, which promotes the root system to undergo profound changes in morphology and architecture. This is accompanied by metabolic reprogramming to maintain intracellular Pi homeostasis.

Two Arabidopsis lncRNAs, *At4* and *IPS1* (*INDUCED BY PHOSPHATE STARVATION 1*) and their orthologous in *Medicago* (*Mt4*) and tomato (*TPS1*) respectively, are strongly induced by Pi starvation. Loss-of-function mutation of *At4* results in defects in Pi redistribution between root and shoot under Pi deficient conditions (Burleigh and Harrison, 1999; Shin et al., 2006). Members of *At4/IPS1* gene family share a conserved 23 nt motif that is partially complementary to *miR399*, but with a central mismatch that prevents miR399 cleavage of the transcript. Hence, binding of *At4/IPS1* lncRNAs acts as a decoy that downregulates *miR399* activity, consequently upregulating its target transcript *PHO2*, which encodes the ubiquitin-conjugating enzyme UBC24 (Figures 1C,E). Targets of UBC24 mediated degradation include several members of the high-affinity phosphate transporters family PHOSPHATE TRANSPORTER1 (PHT1) as well as the PHO1 protein which is involved in Pi loading in the xylem (Liu et al., 2012b, Huang et al., 2013). This molecular mechanism, termed miRNA target mimic (Franco-Zorrilla et al., 2007), is thought to buffer Pi homeostasis under deficient conditions and was until recently the only functionally characterized lncRNA in plants.

Computational evaluation of the *Arabidopsis* and rice genomes identified a large number of putative endogenous miRNA target mimics, of which *miR160* and *miR166* target mimics and their role in *Arabidopsis* development were confirmed (Wu et al., 2013). This suggests lncRNAs may act as miRNA target mimics in a general mechanism of regulation of miRNA activity in plants. This example illustrates that plant lncRNAs influence miRNA mediated gene silencing in addition to their function in transcription and AS. lncRNAs are also involved in translational regulation in plants.

In rice a *cis*-NAT of *OsPHO1;2* (cis-NAT_PHO1;2_) was shown to act as a translational enhancer of *OsPHO1;2* and to contribute to Pi homeostasis (Jabnoune et al., 2013; Figure 1D). *OsPHO1;2* is a functional ortholog of *AtPHO1*, which encodes a protein involved in Pi transport into the root xylem for movement to the shoot (Secco et al., 2010). All three *OsPHO1* orthologs genes have a *cis*-NAT located at the 5′-end of their transcription unit (Secco et al., 2010). Interestingly, the rice *cis-NAT_PHO1;2_* promoter activity is induced by Pi starvation in roots and shoot vascular tissue, whereas *OsPHO1;2* mRNA transcription is constitutive in the same tissues. RNAi knockdown of the rice *cis-NAT_PHO1;2_* caused a decrease in PHO1;2 protein levels, reduced Pi content as well as a decline in yield based on seed weight. By contrast, *cis-NAT_PHO1;2_* overexpression in transgenic rice elevated PHO1;2 levels.

It was hypothesized that *cis*-NAT_PHO1;2_ influences the translation of *OsPHO1;2.* Indeed, the either overexpression of cis-NAT_PHO1;2_ or Pi depletion from the media resulted in association of both *OsPHO1;2* and *cis-NAT_PHO1;2_* RNAs with heavier polysomes, as determined using sucrose density gradients. This indicates that the lncRNA acts to enhance *OsPHO1;2* translation through a mechanism involving association of a lncRNA with polysomal complexes (Figure 1E). Stimulation of translation of a specific mRNA by a lncRNA also occurs in mammalian cells. A *cis*-NAT complementary to mouse *UBIQUITIN CARBOXYTERMINAL HYDROLASE L1* (*Uchl1*) regulates UCHL1 synthesis without changes at the transcript level (Carrieri et al., 2012). These authors identified a short interspersed nuclear element (SINEB2) at the 3′-end of the NAT as necessary for translational enhancement of *Uchl1* mRNA. However, *cis-NAT_PHO1;2_* does not have features of a SINE or other repetitive element. Nonetheless, subcellular transcriptomic analysis in human cell lines revealed a large fraction of lncRNAs is enriched in the cytosol as well as polysomal fractions, whereas only a small subset was found in the nucleus (Van Heesch et al., 2014). Nuclease derived ribosome footprints of *Arabidopsis* included about 10% of the annotated lncRNAs (Juntawong et al., 2014), with most showing no evidence of translating a short open reading frame, raising the possibility that lncRNAs may participate more broadly in translational regulation in plants. Thus, lncRNAs may have a conserved regulatory role in dynamic control of translation of specific transcripts.

## Perspectives

The recent burst of papers describing roles of lncRNAs in plant development and environmentally-regulated developmental plasticity highlights the need for deeper study of the contributions of lncRNA to dynamics of gene expression networks. This is particularly relevant in the control of the root system response to changes in nutrient and water availability where fine-tuning of developmental regulator genes is required to trigger the rapid and reversible developmental and physiological alterations needed to obtain moisture and nutrients.

Functional analysis of lncRNAs is challenging. In many cases, their activity may rely only on secondary structure rather than primary nucleotide sequence. However, the recent advances in genome engineering using designer site-specific nucleases such as CRISPR/Cas9 and TALENs, as well the ability to access to cell-specific and/or subcellular RNA populations using technologies such as isolation of nuclei tagged in specific cell types (INTACT; Deal and Henikoff, 2011) and translating ribosome affinity purification (TRAP; Zanetti et al., 2005; Mustroph et al., 2009), should shed more light on the multiple roles of this “dark matter” of eukaryotic genomes.

### Conflict of Interest Statement

The authors declare that the research was conducted in the absence of any commercial or financial relationships that could be construed as a potential conflict of interest.

## References

[B1] Ben Amor B. Wirth S. Merchan F. Laporte P. d’Aubenton-Carafa Y. Hirsch J. (2009). Novel long non-protein coding RNAs involved in *Arabidopsis* differentiation and stress responses. Genome Res. 19, 57–69. 10.1101/gr.080275.108 18997003 PMC2612962

[B2] Ariel F. Jegu T. Latrasse D. Romero-Barrios N. Christ A. Benhamed M. (2014). Noncoding transcription by alternative RNA polymerases dynamically regulates an auxin-driven chromatin loop. Mol. Cell 55, 383–396. 10.1016/j.molcel.2014.06.011 25018019

[B3] Bardou F. Ariel F. Simpson C. G. Romero-Barrios N. Laporte P. Balzergue S. (2014). Long noncoding RNA modulates alternative splicing regulators in *Arabidopsis*. Dev. Cell 30, 166–176. 10.1016/j.devcel.2014.06.017 25073154

[B4] Birney E. Stamatoyannopoulos J. A. Dutta A. Guigó R. Gingeras T. R. Margulies E. H. (2007). Identification and analysis of functional elements in 1% of the human genome by the ENCODE pilot project. Nature 447, 799–816. 10.1038/nature05874 17571346 PMC2212820

[B5] Burleigh S. H. Harrison M. J. (1999). The down-regulation of Mt4-like genes by phosphate fertilization occurs systemically and involves phosphate translocation to the shoots. Plant Physiol. 119, 241–248. 9880366 10.1104/pp.119.1.241 PMC32226

[B6] Campalans A. Kondorosi A. Crespi M. (2004). Enod40, a short open reading frame-containing mRNA, induces cytoplasmic localization of a nuclear RNA binding protein in *Medicago truncatula*. Plant Cell 16, 1047–1059. 10.1105/tpc.019406 15037734 PMC412876

[B7] Carrieri C. Cimatti L. Biagioli M. Beugnet A. Zucchelli S. Fedele S. (2012). Long non-coding antisense RNA controls Uchl1 translation through an embedded SINEB2 repeat. Nature 491, 454–457. 10.1038/nature11508 23064229

[B8] Cech T. R. Steitz J. A. (2014). The noncoding RNA revolution-trashing old rules to forge new ones. Cell 157, 77–94. 10.1016/j.cell.2014.03.008 24679528

[B9] Deal R. B. Henikoff S. (2011). The INTACT method for cell type-specific gene expression and chromatin profiling in *Arabidopsis thaliana*. Nat. Protoc. 6, 56–68. 10.1038/nprot.2010.175 21212783 PMC7219316

[B10] Franco-Zorrilla J. M. Valli A. Todesco M. Mateos I. Puga M. I. Rubio-Somoza I. (2007). Target mimicry provides a new mechanism for regulation of microRNA activity. Nat. Genet. 39, 1033–1037. 10.1038/ng2079 17643101

[B11] Geisler S. Coller J. (2013). RNA in unexpected places: long non-coding RNA functions in diverse cellular contexts. Nat. Rev. Mol. Cell Biol. 14, 699–712. 10.1038/nrm3679 24105322 PMC4852478

[B12] Van Heesch S. van Iterson M. Jacobi J. Boymans S. Essers P. B. de Bruijn E. (2014). Extensive localization of long noncoding RNAs to the cytosol and mono- and polyribosomal complexes. Genome Biol. 15, R6. 10.1186/gb-2014-15-1-r6 24393600 PMC4053777

[B13] Heo J. B. Sung S. (2011). Vernalization-mediated epigenetic silencing by a long intronic noncoding RNA. Science 331, 76–79. 10.1126/science.1197349 21127216

[B14] Huang F. Zago M. K. Abas L. van Marion A. Galván-Ampudia C. S. Offringa R. (2010). Phosphorylation of conserved PIN motifs directs *Arabidopsis* PIN1 polarity and auxin transport. Plant Cell 22, 1129–1142. 10.1105/tpc.109.072678 20407025 PMC2879764

[B15] Huang T.-K. Han C.-L. Lin S.-I. Chen Y.-J. Tsai Y.-C. Chen Y.-R. (2013). Identification of downstream components of ubiquitin-conjugating enzyme PHOSPHATE2 by quantitative membrane proteomics in *Arabidopsis* roots. Plant Cell 25, 4044–4060. 10.1105/tpc.113.115998 24122829 PMC3877800

[B16] Jabnoune M. Secco D. Lecampion C. Robaglia C. Shu Q. Poirier Y. (2013). A rice cis-natural antisense RNA acts as a translational enhancer for its cognate mRNA and contributes to phosphate homeostasis and plant fitness. Plant Cell 25, 4166–4182. 10.1105/tpc.113.116251 24096344 PMC3877805

[B17] Jiao Y. Meyerowitz E. M. (2010). Cell-type specific analysis of translating RNAs in developing flowers reveals new levels of control. Mol. Syst. Biol. 6:419. 10.1038/msb.2010.76 20924354 PMC2990639

[B18] Juntawong P. Girke T. Bazin J. Bailey-Serres J. (2014). Translational dynamics revealed by genome-wide profiling of ribosome footprints in *Arabidopsis*. Proc. Natl. Acad. Sci. U.S.A. 111, E203–E212. 10.1073/pnas.1317811111 24367078 PMC3890782

[B19] Kestrel, R. and Chen X. (2013). Biogenesis, turnover, and mode of action of plant microRNAs. Plant Cell 25, 2383–2399. 10.1105/tpc.113.113159 23881412 PMC3753372

[B20] Kim E.-D. Sung S. (2012). Long noncoding RNA: unveiling hidden layer of gene regulatory networks. Trends Plant Sci. 17, 16–21. 10.1016/j.tplants.2011.10.008 22104407

[B21] Kim Y. J. Zheng B. Yu Y. Won S. Y. Mo B. Chen X. (2011). The role of Mediator in small and long noncoding RNA production in *Arabidopsis thaliana*. EMBO J. 30, 814–822. 10.1038/emboj.2011.3 21252857 PMC3049218

[B22] Li S. Vandivier L. E. Tu B. Gao L. Won S. Y. li S. (2014). Detection of Pol IV/RDR2-dependent transcripts at the genomic scale in *Arabidopsis* reveals features and regulation of siRNA biogenesis. Genome Res. 25, 235–245. 10.1101/gr.182238.114 25414514 PMC4315297

[B23] Liu J. Jung C. Xu J. Wang H. Deng S. Bernad L. (2012a). Genome-wide analysis uncovers regulation of long intergenic noncoding RNAs in *Arabidopsis*. Plant Cell 24, 4333–4345. 10.1105/tpc.112.102855 23136377 PMC3531837

[B24] Liu T.-Y. Huang T.-K. Tseng C.-Y. Lai Y.-S. Lin S.-I. Lin W.-Y. (2012b). PHO2-dependent degradation of PHO1 modulates phosphate homeostasis in *Arabidopsis*. Plant Cell 24, 2168–2183. 10.1105/tpc.112.096636 22634761 PMC3442594

[B25] Misson J. Raghothama K. G. Jain A. Jouhet J. Block M. A. Bligny R. (2005). A genome-wide transcriptional analysis using *Arabidopsis thaliana* Affymetrix gene chips determined plant responses to phosphate deprivation. Proc. Natl. Acad. Sci. U.S.A. 102, 11934–11939. 10.1073/pnas.0505266102 16085708 PMC1188001

[B26] Mustroph A. Zanetti M. E. Jang C. J. H. Holtan H. E. Repetti P. P. Galbraith D. W. (2009). Profiling translatomes of discrete cell populations resolves altered cellular priorities during hypoxia in *Arabidopsis*. Proc. Natl. Acad. Sci. U.S.A. 106, 18843–18848. 10.1073/pnas.0906131106 19843695 PMC2764735

[B27] Rinn J. L. Chang H. Y. (2012). Genome regulation by long noncoding RNAs. Annu. Rev. Biochem. 81, 145–166. 10.1146/annurev-biochem-051410-092902 22663078 PMC3858397

[B28] Secco D. Baumann A. Poirier Y. (2010). Characterization of the rice PHO1 gene family reveals a key role for OsPHO1;2 in phosphate homeostasis and the evolution of a distinct clade in dicotyledons. Plant Physiol. 152, 1693–1704. 10.1104/pp.109.149872 20081045 PMC2832267

[B29] Secco D. Jabnoune M. Walker H. Shou H. Wu P. Poirier Y. (2013). Spatio-temporal transcript profiling of rice roots and shoots in response to phosphate starvation and recovery. Plant Cell 25, 4285–4304. 10.1105/tpc.113.117325 24249833 PMC3875719

[B30] Shin H. Shin H.-S. Chen R. Harrison M. J. (2006). Loss of At4 function impacts phosphate distribution between the roots and the shoots during phosphate starvation. Plant J. 45, 712–726. 10.1111/j.1365-313X.2005.02629.x 16460506

[B31] Sugiyama T. Cam H. P. Sugiyama R. Noma K. Zofall M. Kobayashi R. (2007). SHREC, an effector complex for heterochromatic transcriptional silencing. Cell 128, 491–504. 10.1016/j.cell.2006.12.035 17289569

[B32] Swiezewski S. Liu F. Magusin A. Dean C. (2009). Cold-induced silencing by long antisense transcripts of an *Arabidopsis* Polycomb target. Nature 462, 799–802. 10.1038/nature08618 20010688

[B33] Vaughn M. W. Martienssen R. A. (2005). Finding the right template: RNA Pol IV, a plant-specific RNA polymerase. Mol. Cell 17, 754–756. 10.1016/j.molcel.2005.03.003 15780931

[B34] Wang H. Chung P. J. Liu J. Jang I.-C. Kean M. J. Xu J. (2014). Genome-wide identification of long noncoding natural antisense transcripts and their responses to light in *Arabidopsis*. Genome Res. 24, 444–453. 10.1101/gr.165555.113 24402519 PMC3941109

[B35] Wierzbicki A. T. Haag J. R. Pikaard C. S. (2008). Noncoding transcription by RNA polymerase Pol IVb/Pol V mediates transcriptional silencing of overlapping and adjacent genes. Cell 135, 635–648. 10.1016/j.cell.2008.09.035 19013275 PMC2602798

[B36] Wu H.-J. Wang Z.-M. Wang M. Wang X.-J. (2013). Widespread long noncoding RNAs as endogenous target mimics for microRNAs in plants. Plant Physiol. 161, 1875–1884. 10.1104/pp.113.215962 23429259 PMC3613462

[B37] Zanetti M. E. Chang I.-F. Gong F. Galbraith D. W. Bailey-Serres J. (2005). Immunopurification of polyribosomal complexes of *Arabidopsis* for global analysis of gene expression. Plant Physiol. 138, 624–635. 10.1104/pp.105.059477 15955926 PMC1150383

[B38] Zhang X. Henderson I. R. Lu C. Green P. J. Jacobsen S. E. (2007). Role of RNA polymerase IV in plant small RNA metabolism. Proc. Natl. Acad. Sci. U.S.A. 104, 4536–4541. 10.1073/pnas.0611456104 17360559 PMC1810338

